# Clinical utility of 123I‐Ioflupane SPECT striatal binding for evaluating parkinsonism: a retrospective study of a dementia with Lewy bodies cohort

**DOI:** 10.1002/alz.093889

**Published:** 2025-01-09

**Authors:** Emily F Maly, James B Leverenz, Brittany Lapin, Yadi Li, Frank A DiFilippo

**Affiliations:** ^1^ Cleveland Clinic, Cleveland, OH USA

## Abstract

**Background:**

This study set to determine if 123I‐ioflupane SPECT striatal binding ratio (SBR) correlated with parkinsonian symptoms measured on Movement Disorder Society – Unified Parkinson’s Disease Rating Scale Part III (UPDRS‐III) in a dementia with Lewy bodies (DLB) cohort and if SBR measured at baseline could predict progression of parkinsonian symptoms over 2 years.

**Methods:**

This is a retrospective cohort study using the U.S. Dementia with Lewy Bodies Consortium (DLBC) dataset. Sample group included 54 individuals with probable DLB or mild cognitive impairment with Lewy bodies (MCI‐LB). Participants had baseline 123I‐ioflupane SPECT scan analyzed with DaTQUANT and baseline UPDRS‐III examination; a subset of 25 participants had a 24‐month UPDRS‐III examination. Mean of the left and right SBR Z‐scores for the striatum, putamen, posterior putamen, and caudate were analyzed. A subset of 45 participants had CSF tested with [Amprion] a‐synulcein seed amplification assay (SAA).

**Results:**

Multivariable linear regression models were used to predict 54 participants’ UPDRS‐III at baseline; this showed baseline SBRs are significant predictors of baseline UPDRS‐III scores with the caudate SBR having the strongest correlation (p=0.005). Multivariable linear regression models were used to predict 25 participants’ UPDRS‐III score at 24‐months; this showed baseline SBRs are significant predictors of 24‐month UPDRS‐III scores with the caudate SBR having the strongest correlation (p=0.009). When adjusting for baseline UPDRS‐III scores, SBRs are no longer significant predictors of 24‐month UPDRS‐III scores (p=0.36). When a multivariable logistic regression model was used to predict meaningful worsening in UPDRS‐III from baseline to 24‐months (defined as worsening by = +5 points) the relationship was not significant (AUC=0.53). A sub‐analysis of the 45 participants with a‐synulcein SAA showed that presence a‐synulcein was associated with higher UPDRS‐III scores and lower SBRs.

**Conclusion:**

Findings reveal a significant relationship between 123I‐ioflupane SPECT SBRs and motor parkinsonism in DLB as measured on UPDRS‐III. In DLB, 123I‐ioflupane SPECT scan, at time of diagnosis, could be used to confirm underlying dopamine deficiency in the context of clinical parkinsonism. Findings suggest baseline 123I‐ioflupane SPECT SBRs do not add value beyond baseline UPDRS‐III in predicting clinical progression of parkinsonism in 2 years.

